# Multidisciplinary team for patients with neurocutaneous syndromes: The little discussed importance of dentistry

**DOI:** 10.1016/j.clinsp.2024.100332

**Published:** 2024-02-15

**Authors:** Marcos Roberto Tovani-Palone, Filippo Bistagnino, Pritik A. Shah

**Affiliations:** a Department of Research Analytics, Saveetha Dental College and Hospitals, Saveetha Institute of Medical and Technical Sciences (SIMATS), Chennai, India; b Department of Medical Biotechnology and Translational Medicine, International Medical School, Università degli Studi di Milano, Milan, Italy; c Bangalore Medical College and Research Institute, Bangalore, India

**Keywords:** Dentistry, Neurocutaneous syndromes, Patient care team, Delivery of health care, Comprehensive health care

## Abstract

Neurocutaneous syndromes comprise a heterogeneous group of congenital or hereditary conditions that are known to be associated with the risk of different disorders and complications. Two of the most common neurocutaneous syndromes are Neurofibromatosis type 1 (NF1) and Tuberous Sclerosis Complex (TSC). Although there appears to be a general consensus on the importance of a multidisciplinary approach in managing these cases, there is still very little emphasis in discussions addressed in the literature on the role of dentistry in accordance with the perspective of comprehensive care. Evidence-based propositions, together with a broad discussion of new insights in this regard, should have the ability to strongly impact related future perspectives, aiming for greater advances and better outcomes for these patients. In this review article, the authors discuss updated general aspects of NF1 and TSC, and the potential additional roles of dentistry, in addition to addressing suggestions for actions in dentistry at related levels of care, as well as priorities for future research.

## Highlights

• The inclusion of dentists in multidisciplinary teams for patients with neurocutaneous syndromes is essential, both on an outpatient and hospital basis.
• Interestingly, dentistry can play a relevant role in the early diagnosis and differential diagnosis of patients with neurocutaneous syndromes.
• More research in this area is needed.

## Background

An efficient and effective integration between medicine and other health professions is still a major challenge to be overcome by multidisciplinary teams around the world. This is true, especially for the management of less frequent conditions within a group of diseases that require more complex care, as is the case with neurocutaneous syndromes. In this sense, the literature has emphatically highlighted the importance of involving different medical specialties in the multidisciplinary approach to such patients.^1^^,^^2^ However, thinking about holistic care and better outcomes, other health fields and in particular dentistry should also be essential to be included as part of such teams. Although the role and difficulties faced by dentistry in the management of patients with the most prevalent neurological disorders have already been addressed in the literature,^3^^,^^4^ there is still very little discussion involving specific subgroups. In this brief review the authors discuss general aspects of neurocutaneous syndromes, the potential additional role of dentistry and its benefits in affected patients, with a special focus on Neurofibromatosis type 1 (NF1 [OMIM 162200]) and Tuberous Sclerosis Complex (TSC [OMIM 191100/613254]), in addition to proposing suggestions for actions in dentistry at related levels of care and for further research.

### General aspects of Neurofibromatosis type 1 and Tuberous Sclerosis Complex

Neurocutaneous syndromes, also known as phakomatoses, comprise a broad group of congenital or hereditary conditions that affect, among other organs, the skin and nervous system. Among the diseases within this classification, the two most common are Neurofibromatosis type 1 (NF1) and Tuberous Sclerosis Complex (TSC).^1^^,^^5^

### Neurofibromatosis type 1

Neurofibromatosis type 1 (NF1) is defined from a genetic perspective as an inherited or non-inherited disease caused in the first case by an autosomal dominant mutation in the *NF1* gene, which has a tumor-suppressive function. This gene encodes the protein neurofibromin that acts as a negative regulator of the Ras signaling pathway, responsible for controlling cell proliferation.^6^ Evidence indicates that around half of the patients can have sporadic cases associated with a new (or de novo) mutation in the respective gene.^7^

Based on its clinical presentations, NF1 usually presents as a multisystem disorder that mainly affects the skin, nervous system, eyes, and bones, implying a greater tendency to develop neoplasms at younger ages. In this context, a worse outcome compared to the general population together with an estimated decrease in life expectancy of up to 15 years may be expected.^8^^,^^9^ Among the most common clinical manifestations are pigmentary abnormalities like café-au-lait macules, freckles in the axillary and inguinal region (found in about 96.5 % and 90 % of cases, respectively), and iris Lisch nodules (found in 41–86 % of cases).^10^^,^^11^ Another important finding is the presence of neurofibromas, which are benign Schwann cell tumors. Among their subtypes are cutaneous and subcutaneous neurofibromas (benign tumors), and plexiform neurofibromas, which despite being a significant cause of complications, are often inoperable due to their invasiveness, with the potential to transform into malignant peripheral nerve sheath tumors.^7^^,^^12^ Malignant tumors, including those of the optic pathways and brainstem gliomas, are present in 15–20 % of NF1-affected patients.^3^

Skeletal deformities like scoliosis (found in 10–26 % of cases), sphenoid wing dysplasia, congenital tibial dysplasia, and pseudoarthrosis, in addition to osteopenia, are among other possible presentations that should be taken into consideration in the diagnosis of NF1, as shown in Table 1. It is also very common for these patients to present neurocognitive changes demonstrated as learning difficulties, which are present in around 50 % of patients.^2^^,^^7^

**Table 1:** Main highlights related to oral findings within the diagnostic criteria for Neurofibromatosis type 1 and Tuberous Sclerosis Complex.

Source: Adapted from Legius et al^23^ and Northrup et al.^24^

### Tuberous Sclerosis Complex

Tuberous Sclerosis Complex (TSC) is also another multisystem condition. Its etiology is related to two mutations involving the *TSC1* and *TSC2* genes, which are responsible for regulating the mammalian Target of Rapamycin (mTOR) molecule, causing it to become hyperactivated. The related mutations can either occur de novo (in two-thirds of the cases) or be inherited.^13^ As a result, tumorigenesis may be observed, as well as neurological and behavioral abnormalities, in response to the role of mTOR in controlling cellular growth, proliferation, and translational mechanisms. In this connection, cellular hyperplasia and dysplasia may be present. It is worth highlighting that such genes encode for two proteins, hamartin (the product of the *TSC1* gene) and tuberin (the product of the *TSC2* gene), both involved in the inhibition of the mTOR signaling cascade.^14^

The most important clinical manifestations resulting from this deregulated activation mainly affect the central nervous system. Most of patients (90 % of cases) are affected by epilepsy, cortical tubers (80–90 % of cases), and subependymal nodules (80 % of cases). Furthermore, other cognitive impairments like autism spectrum disorders and intellectual disability can also be present.^14^ Another important manifestation that is also related to the high morbidity and mortality of the disease is the occurrence of renal abnormalities (found in 60–80 % of patients). In these cases, different types of renal lesions can occur including renal angiomyolipoma (although benign, it has a high risk of bleeding), single or multiple cysts, and renal cell carcinoma.^13^, ^15^ As it is a multisystemic disease, many other less prevalent changes can be observed in affected patients and should not be neglected by TSC healthcare teams, such as the case of gingival fibromas that can be present in up to 20 % of patients.^14^ Other possible lesions are listed in Table 1 and are part of the TSC diagnostic criteria.

### Public health estimates for both conditions

Although both conditions here addressed are not among the most common neurological diseases, their burden may have significant impacts from a public health perspective, making early diagnosis and treatment essential. According to the literature, Neurofibromatosis type 1 (NF1) has an estimated prevalence of 1:3000 live births (it is the most common neurocutaneous syndrome), while that of Tuberous Sclerosis Complex (TSC) is approximately 1:6000.^5^

Another highlight concerns the related mortality rates. A considerably higher mortality risk has been reported in evidence-based studies involving patients affected by NF1 compared to the general population, which has been mainly attributed to the possible malignant transformation of NF1 tumors. This in turn is corroborated by the cohort study conducted by Duong et al., these authors evaluated data from the French population and found a noticeable increase in overall mortality due to the disease (with a standardized mortality ratio of 2.2).^16^

A similar situation has been verified for TSC. The findings of an important study conducted in Taiwan that evaluated the cumulative mortality of TSC, obtained through analysis of Kaplan-Meier curves from enrollment (diagnosis), found rates of approximately 4 % after 7 years. Moreover, higher rates were observed for patients diagnosed after the age of 18 (9.94 % vs. 1.82 %), suggesting that age enrollment may actually be a relevant prognostic factor^17^ while highlighting the importance of early multidisciplinary pediatric care. On that same occasion, these authors^17^ also evaluated the standardized mortality ratio, comparing the cumulative mortality in patients with TSC with the general population (during the same study period), and found a mortality rate of 4.9, which was very similar to that obtained by a previous study conducted in the Caucasian population (more specifically in the Netherlands) whose value was found to be 4.8. Such rates have been mainly related to the presence of renal disease,^15^^,^^17^ including renal angiomyolipoma and other kidney complications like renal angiomyolipoma abscess.^15^

In reaction to the above, further healthcare actions aimed at mitigating such mortality rates are equally imminent to be effectively achieved within a global context considering the data already available and several challenges to be overcome. In this way, multidisciplinary teams made up of doctors, nurses, geneticists, physiotherapists, psychologists, among other health professionals, including dentists, are essential for this purpose. This last item involving dentistry is the subject of the present discussion below.

### Dental care and neurocutaneous syndromes

In addition to individual clinical experience, recent and robust evidence and reports of institutional experience have suggested different positive impacts of specialized dental care for neurological patients, whether in an outpatient^3^ or hospital setting^4^ In the context of patients with neurocutaneous syndromes, such benefits may be impactful as related conditions requiring this type of specialized management can be alternatively or concomitantly present, including and not only restricted to craniofacial defects, dental and oral anomalies but also neurocognitive alterations.^1^ Based on these perspectives, dentistry can play an important role in improving the quality of life, contributing to the prevention of further damage, while allowing a faster recovery with better outcomes.^4^^,^^18^^,^^19^

### Outpatient and hospital levels

At the outpatient level, the simultaneous presence of neurocognitive disabilities in these patients should always be taken into consideration in the management of oral healthcare. Indeed, apart from the more common expected complications like caries lesion, periodontal and endodontic problems,^4^ other more specific associated disorders and anomalies including important occlusal problems,^20^ and intraoral fibromas and dental enamel pits^1^^,^^21^ (which are among the criteria for diagnosis of Neurofibromatosis type 1 and Tuberous Sclerosis Complex (TSC) – Table 1) may increase the likelihood for patients with neurocutaneous syndromes to autonomously perform inadequate oral hygiene, exposing them excessively to unnecessary and preventable risks.

The importance of more thorough oral care for these patients has been the subject of previous discussion in the literature. Teng et al. reinforced in their international consensus statement the crucial role of early intervention by dental specialists to prevent further complications for patients with TSC, in which the impact of intellectual disabilities and behavioral issues that can make oral hygiene extremely difficult can reach up to 50 % of cases.^22^ Dentists are hence clearly an essential, but often absent, figure in multidisciplinary teams for patients with neurocutaneous syndromes.

Moreover, all the oral conditions previously mentioned might represent an increased risk for the development of postoperative infections in the case these patients undergo a surgical procedure-particularly involving the craniomaxillofacial region, as well as for the occurrence of other related complications. Therefore, in these circumstances, it should be of paramount importance to prevent any infectious foci in the oral cavity before and after required surgical procedures,^4^^,^^18^ given that the oral cavity is colonized by a wide range of different species of bacteria, fungi, and viruses, comprising almost half of the members of the human microbiota.^18^

**Fig. 1:**
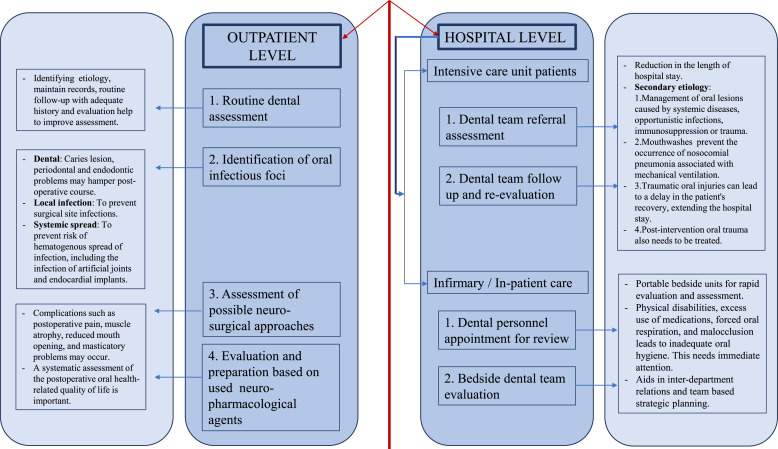
Proposal for comprehensive oral care at different levels of care for patients with neurocutaneous syndromes. Source: Adapted from Rebelo M et al.,^1^ Galeotti e al. ^4^ Palone,^18^ Tovani-Palone et al.,^19^ Korporowicz et al.,^21^ Teng et al.,^22^ Coll et al.,^25^ Saleh et al.^26^

This may have an increased impact on more severe cases requiring surgery and hospitalization.^1^ At the hospital level, the dental assessment and/or dental follow-up of hospitalized patients is essential, especially in intensive care units.^19^ Based on the advances of dentistry aimed at the specialized care for patients with special needs, which includes neurological patients, the authors created a flowchart of a schematic representation of the different levels of dental practice towards the comprehensive care of patients with neurocutaneous syndromes (Fig. 1), summarizing here the authors’ suggestions.

### The role of dentistry in the early diagnosis

A further relevant point to be addressed within the present discussion is that both at the hospital and outpatient setting, specialized dentists can play a crucial role not only in terms of complication prevention, as already mentioned, but also in terms of anticipating the diagnosis of such syndromes.

### Focus on Neurofibromatosis type 1

In two interesting case reports^27^^,^^28^ the importance of such contribution for cases of Neurofibromatosis type 1 (NF1) is highlighted. In these manuscripts, different reports are presented and the common denominator between them is that the disease was specifically diagnosed during dental consultations. In one of them^27^ the patient's main complaint was related to the presence of a slowly growing mass at the mandibular level, subsequently identified as a neurofibroma, together with other typical NF1 manifestations, including café au lait spots on the trunk, shin and knees, and subcutaneous neurofibromas. Oral manifestations and lesions can be present in up to 72 % of patients affected by NF1,^28^^,^^29^ and among the most common presentations are enlargement of the fungiform papillae (in 50 % of cases), presence of single or multiple neurofibromas on hard or soft oral tissues, wide inferior alveolar canals, and enlarged mandibular foramina.^27^, ^28^, ^29^ It is worth highlighting here that the presence of neurofibromas, in addition to their individual implications, can also be associated with the occurrence of different dental, buccal, and maxillofacial disorders like tooth mobility, eruption dysfunction, inclusion or infraocclusion of deciduous or permanent teeth, and hypertrophy of underlying bone structures.^30^

Another possible peculiar feature is highlighted in a descriptive analysis by Friedrich et al., who observed the occurrence of aplasia in lower second molars in four of their 26 patients with NF1 affected by plexiform neurofibroma. This finding was furthermore associated with a distal position of the first molar and hypoplasia of the ipsilateral mandible. In all these four cases were verified the presence of a plexiform neurofibroma at the level of the second and third trigeminal branches, adjacent to the ipsilateral alveolar ridge.^31^ More in-depth investigations on this topic may be interesting to conduct in order to better understand the existence of a possible relationship between the occurrence of oral plexiform neurofibromas and their influence on the development of orofacial malformations, which should benefit an eventual implementation of new parameters within the diagnostic criteria for NF1.

### Focus on Tuberous Sclerosis Complex

Among the findings of dental interest in patients with Tuberous Sclerosis Complex (TSC), intraoral fibromas and dental enamel pits stand out, which, as previously mentioned, are listed among the diagnostic criteria for the disease (Table 1). According to literature estimates, the occurrence of oral fibromas in patients with TSC has a variable incidence, affecting approximately 36 % to 69 % of cases.^32^ Such variation may be associated with the age of the population considered. Furthermore, the most commonly affected oral region is the gingival region.^32^^,^^33^ This is so true that in the 1998 consensus criteria only gingival fibroma had been included within the minor criteria related to oral findings.^22^

Not least, dental enamel pits are present in a high percentage of patients with TSC. Although such dental anomaly is also observed in the general population, its prevalence and number of lesions are generally much lower compared to the cases of TSC.^22^, ^34^, ^35^ As a result, oral neurofibromas and dental enamel pits constitute key features to be clinically detected by dentists during oral assessment, with the potential to substantially contribute and/or assist towards establishing an early diagnosis for TSC.

### Related recommendations for multidisciplinary teams

First, all these additional manifestations or lesions involving cases of NF1 and TSC should be accurately identified and/or managed. In this connection, an interesting point to be emphasized and taken into account is that the more specific oral findings of both conditions should not be negligible in terms of frequency, given that they can occur together with a series of other disorders, including neurocognitive ones, with the possibility to predispose to a deterioration in the general condition of these patients. Second, this is one more reason why it is of paramount importance and essential to support the presence of competent and specialized personnel within the multidisciplinary teams involved in the treatment and follow-up of patients with neurocutaneous syndromes. Achieving this goal would benefit the patients both in terms of earlier diagnosis and disease management.

### The role of dentistry in differential diagnosis

Dentists can also play another prominent role within this scope, contributing to the establishment of differential diagnoses. In a study by Kobayashi, Matsune, and Ohashi, the occurrence of distinctive oral manifestations for cases of Neurofibromatosis type 1 (NF1) with *NF1* gene deletion compared to *NF1* gene mutation was clearly demonstrated. Among these manifestations is the presence of fused teeth, evident macrodontia, and increased dental caries in patients with *NF1* deletion.^36^ Such authors therefore found important evidence to assist in the clinical differential diagnosis of different subtypes of NF1, supporting in particular the work of geneticists. Furthermore, this should also be of great use for the development of more specific treatment protocols, as well as to lead to earlier and more assertive interventions.

### Research priorities in the area of dentistry for patients with neurocutaneous syndromes

Through a quick search in the PubMed and Scopus databases, using the following keywords and Boolean operators’ “dentistry” AND “tuberous sclerosis complex” or “dentistry” AND “neurofibromatosis type 1”, it is possible to highlight the existence of considerable scarcity of studies in the international peer-reviewed literature about comprehensive oral treatment for patients with neurocutaneous syndromes, as well as about the current related situation. Being aware of the importance and real impacts of more research in this area, the authors have outlined six research priorities (Table 2).

**Table 2:** Research priorities in dentistry for patients with neurocutaneous syndromes.

1. Determine the dental public health status related to these patients at the global, national, and local levels
2. Development and implementation of new specialized approaches to increase the effectiveness of dental management for this target audience
3. Determine the effects of neurocutaneous syndromes on craniofacial development in different populations
4. Determine the effects of neurocutaneous syndromes on dental development in different populations
5. Elucidate the oral microbiota composition of affected patients
6. Measuring the impact of dentistry on the treatment and quality of life of these patients

### Final consideration

Finally, much more than multidisciplinary teams, greater engagement towards interdisciplinary and even transdisciplinary assessments, aiming at deeper integration of medicine with other allied health professions,^37^^,^^38^ could function as a key element for further advances and better outcomes for these patients. Moreover, increased efforts are imminently needed to be taken to enhance clinical training and provide more in-depth knowledge in neurocutaneous syndromes, especially among dentists in the areas of patients with special needs and/or hospital dentistry in order to achieve specialized training that enables more comprehensive and sustained attention to the assessment of individual patients, along with greater confidence in patient and/or caregiver education and collaboration. All of these aspirations should, without a doubt, be positively impacted by more research in the area, which is strongly recommended and encouraged by the authors.

## Funding

This research received no specific grant from any funding agency in the public, commercial, or not-for-profit sectors.

## CRediT authorship contribution statement

**Marcos Roberto Tovani-Palone:** Conceptualization, Writing – original draft, Supervision. **Filippo Bistagnino:** Conceptualization, Writing – original draft, Writing – review & editing. **Pritik A. Shah:** Writing – original draft, Writing – review & editing.

## Declaration of competing interest

The authors declare no conflicts of interest.
